# Supplementary material for: ChIP-Hub provides an integrative platform for exploring plant regulome
Source: Nat Commun. 2022 Jun 14;13:3413. doi: 10.1038/s41467-022-30770-1 (PMC9197862; doi:10.1038/s41467-022-30770-1)
Supplement: Supplementary file 1 — Supplementary Information [file 41467_2022_30770_MOESM1_ESM.pdf]

ChIP-Hub provides an integrative platform for exploring plant regulome

Fu *et al.*

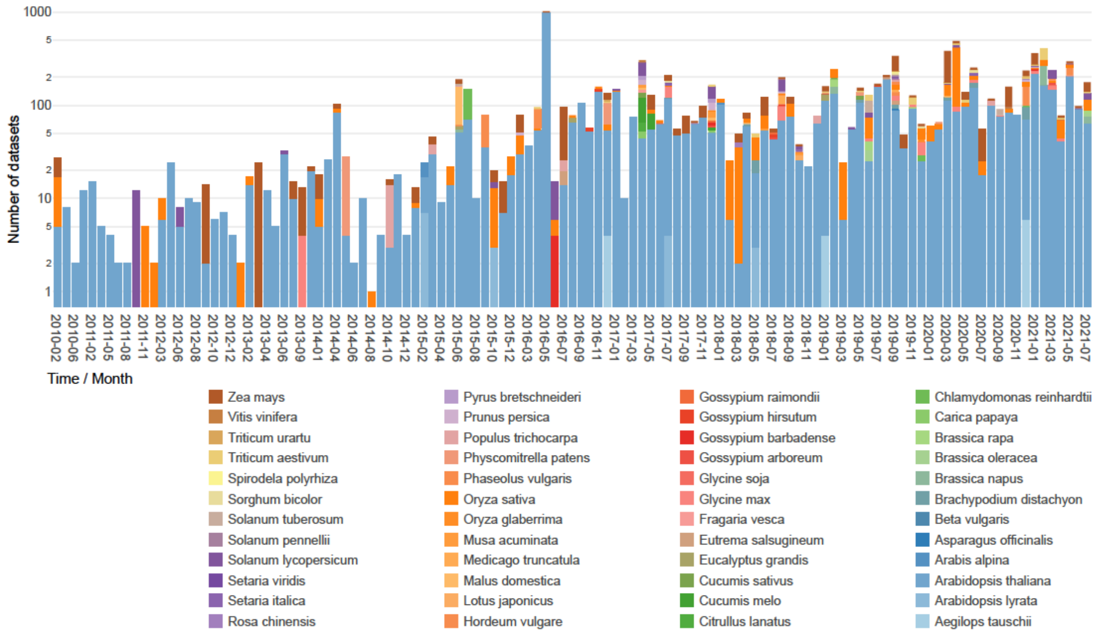

Supplementary Fig. 1. Statistics of publicly available regulome datasets in plants. Barchart showing the release of plant datasets in the past years. Datasets are colored by different plant species.

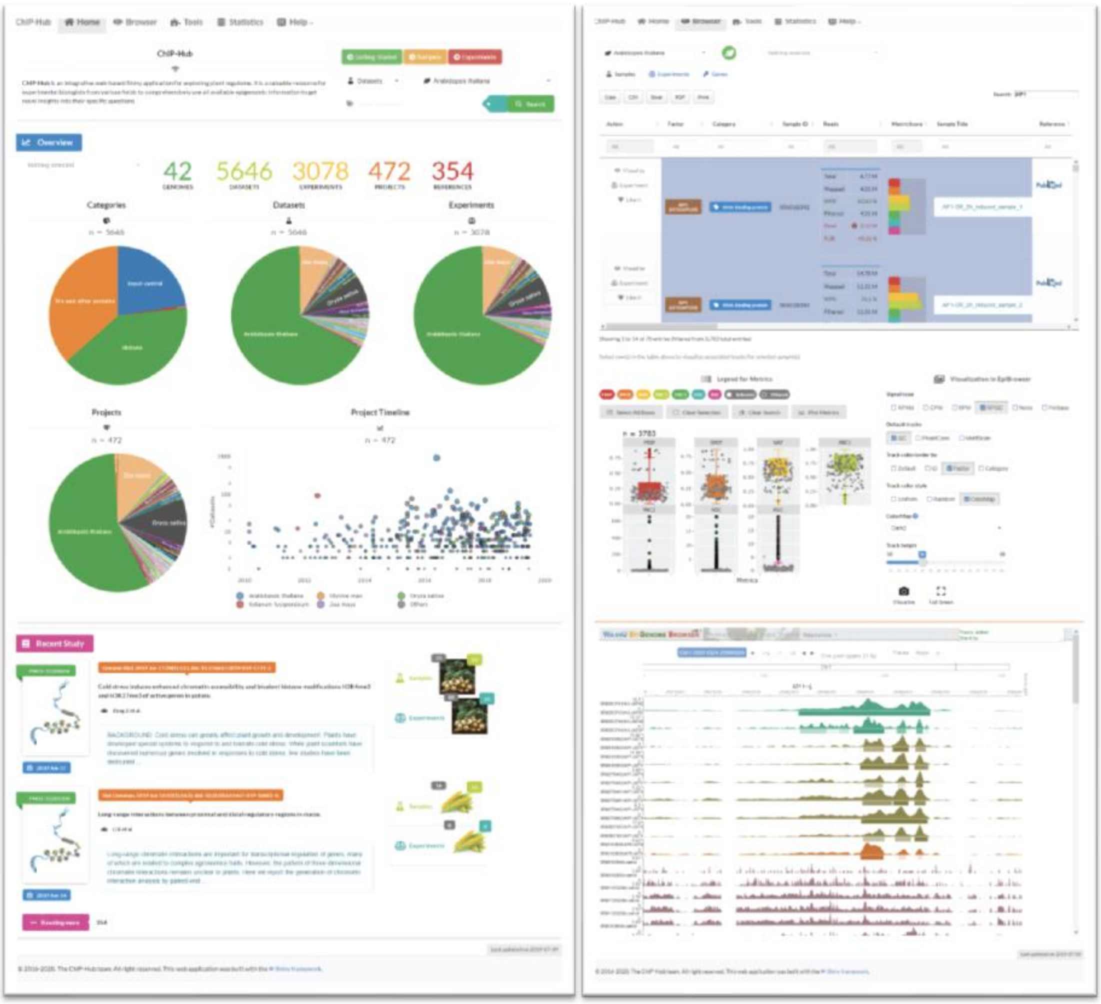

Supplementary Fig. 2. The ChIP-Hub Shiny application. Screenshots of the ChIP-Hub website. The left panel shows the "Home" page of ChIP-Hub. The right panel shows the "Browser" page with example tracks of ChIP-seq datasets shown at the bottom genome browser. Please visit our website (https://biobigdata.nju.edu.cn/ChIPHub/) for interactive pages.

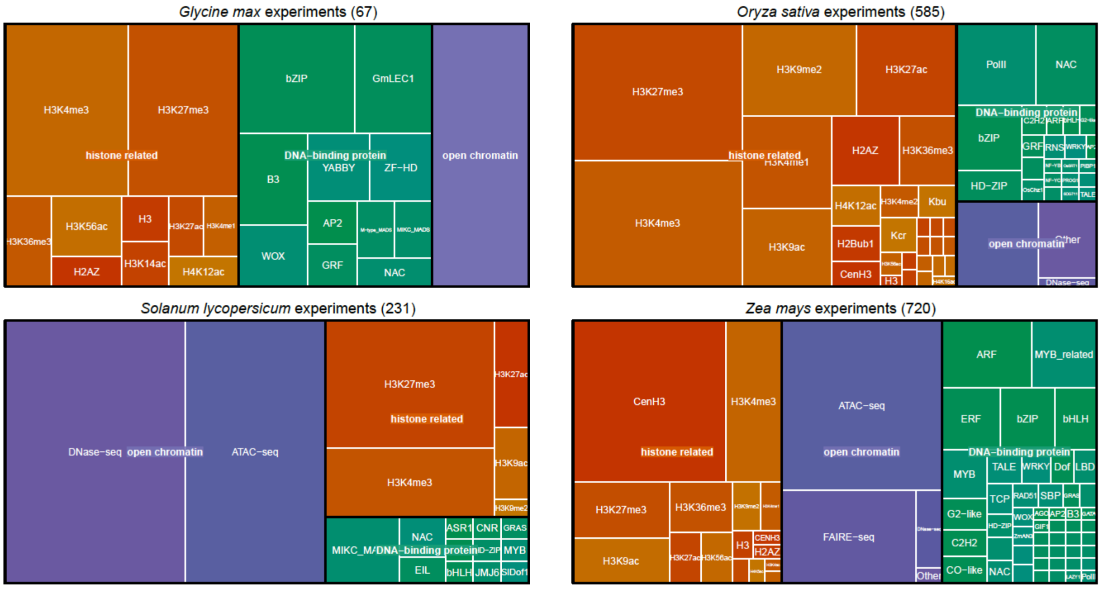

Supplementary Fig. 3. Examples of metadata files. Treemap showing the classification of experiments in *Glycine max*, (*Oryza sativa*), (*Solanum lycopersicum*), and (*Zea mays*), according to transcription factor (TF) families, the types of histone modifications or open chromatin experiments.

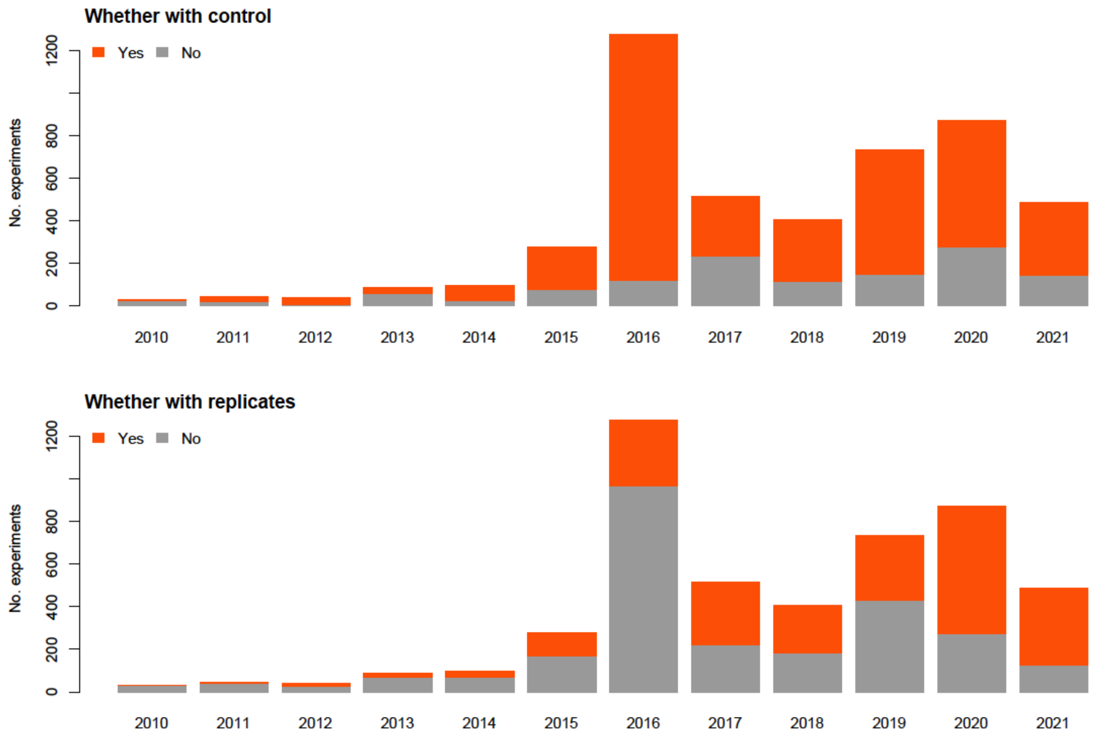

Supplementary Fig. 4. Summary of the quality of experiments according to the time. Barcharts showing whether the experiments with proper input control or with replicates.

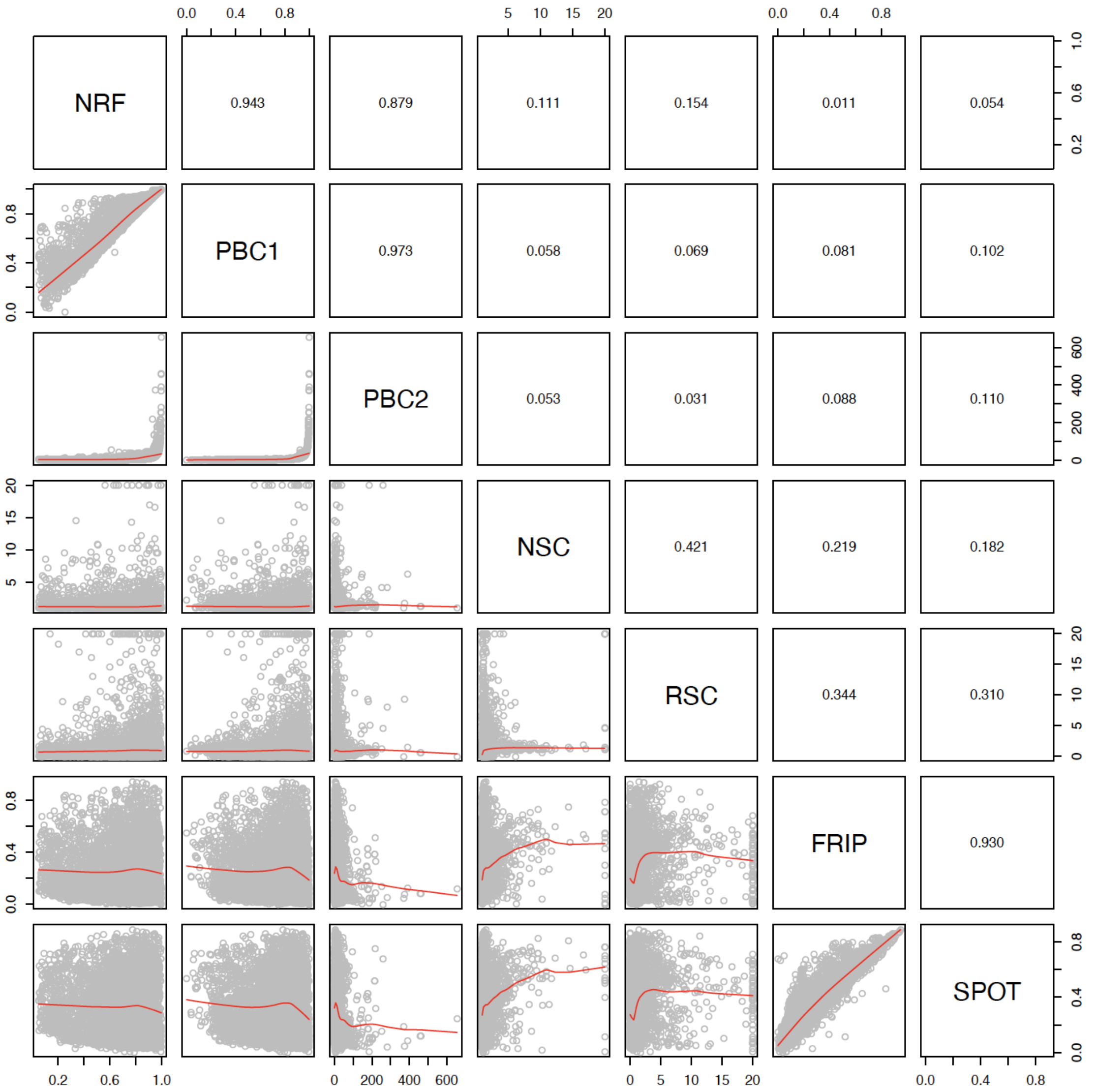

Supplementary Fig. 5. Correlation of metrics scores. Scatter plots (the lower panel) showing the Pearson's correlation coefficients (the upper panel) of different metrics. SPOT: signal portion of tags; FRiP: fraction of reads in peaks; NSC: normalized strand cross-correlation coefficient. RSC: relative Strand cross-correlation coefficient; NRF: non-redundant fraction; PBC1/2: PCR bottlenecking coefficients 1/2.

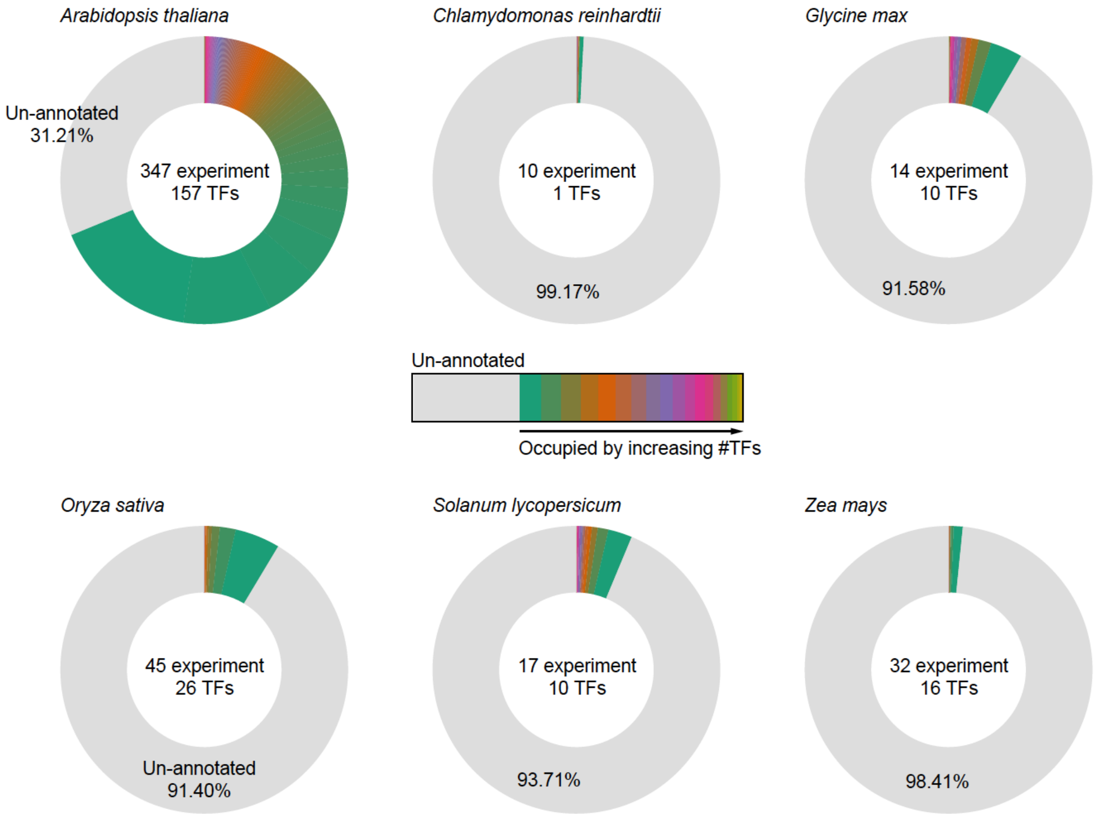

Supplementary Fig. 6. Fraction of plant genomes occupied by transcription factor (TF) binding sites (TFBSs). Donut charts showing the fraction of the entire genome covered by TFBSs based on ChIP-seq data in the selected plant species. The number of ChIP-seq experiments and the associated distinct TFs are indicated. The genome is colored according to the number of occupied TFs.

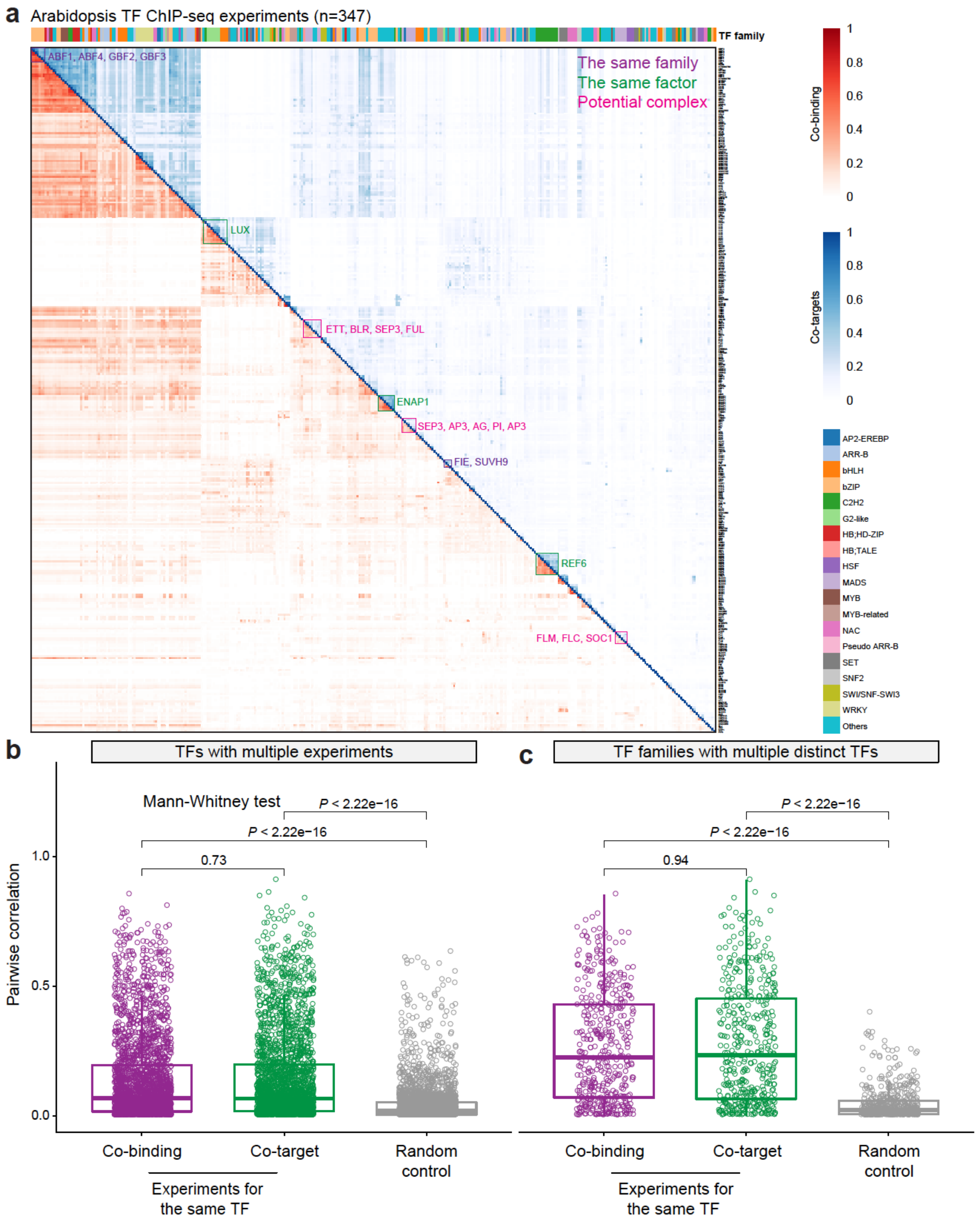

Supplementary Fig. 7. Co-associations of TFs in *Arabidopsis thaliana*, related to Fig. 3. (a) Heatmap showing co-binding relationships (upper triangle) and co-regulated targets (lower) by TFs. Each row or column represents one TF in a specific ChIP-seq experiment. Co-associations were both calculated based on Jaccard statistics with either peak basepair or target genes. TFs from different families were colored in the left bar. In general, the same TF in different experiments and TFs from the same family or from known protein complexes showed significantly higher associations than random controls (Supplementary Fig. 9). For example, MADS-domain TFs that act in a combinatorial manner in specifying floral organ

identities (such as SEP3, AP3, AP1, AG and PI)[1] or developmental phase transitions (such as FLC, FLM, and SOC1)[2–4] strongly overlap in their DNA-binding sites. Boxplots (individual data points as overlays) showing that the same TF with multiple experiments **(b)** or TFs from the same family **(c)** have significantly higher associations than random controls. As random control, the same number of associations were sampled from the rest of the comparisons. Statistical significance of difference was calculated by the two-sided Mann-Whitney U test. Boxplot shows the median (horizontal line), second to third quartiles (box), and Tukey-style whiskers (beyond the box).

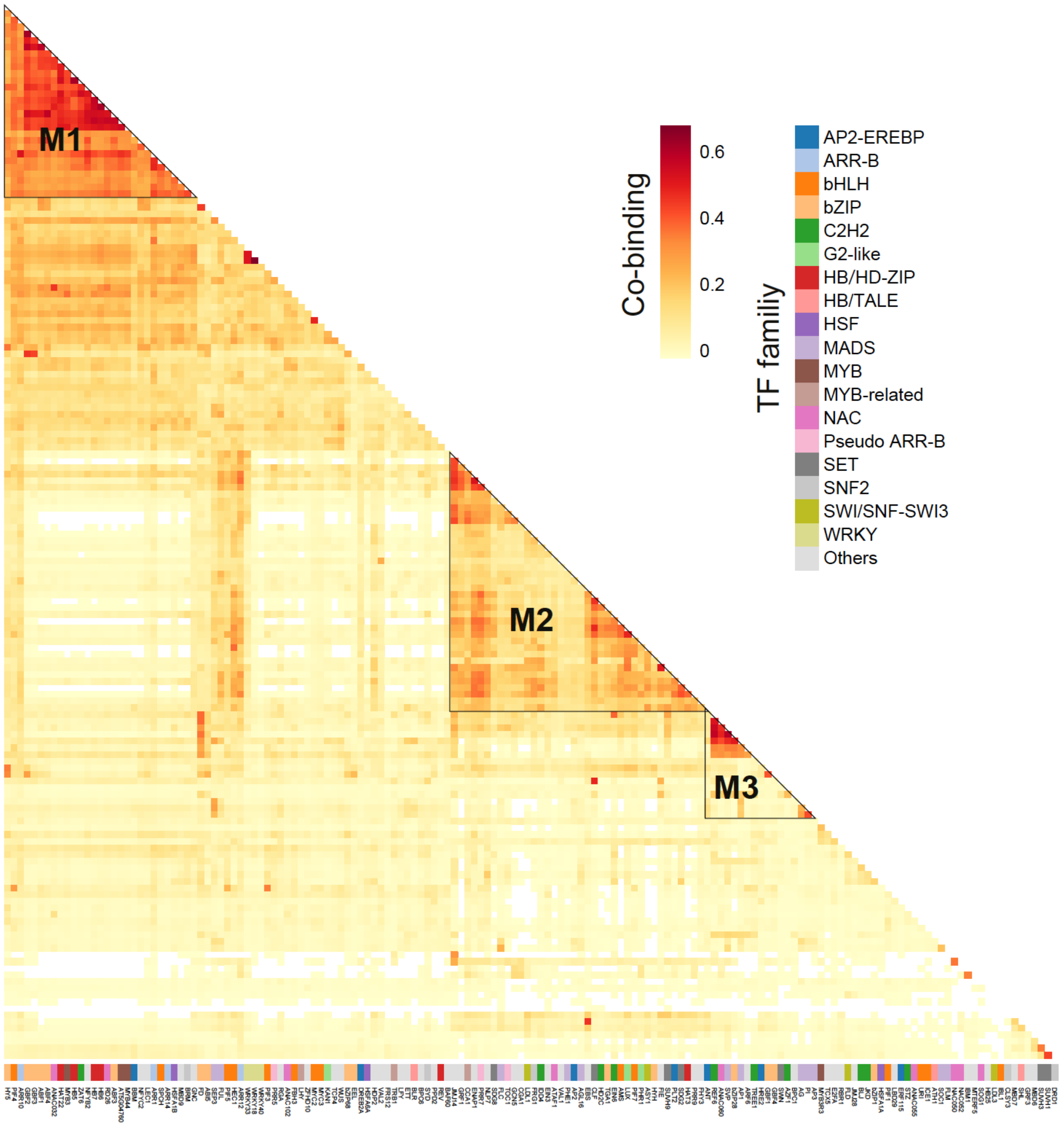

Supplementary Fig. 8. Co-associations of TFs, related to Fig. 3. Heatmap showing co-binding relationships of all investigated TFs (n=157).

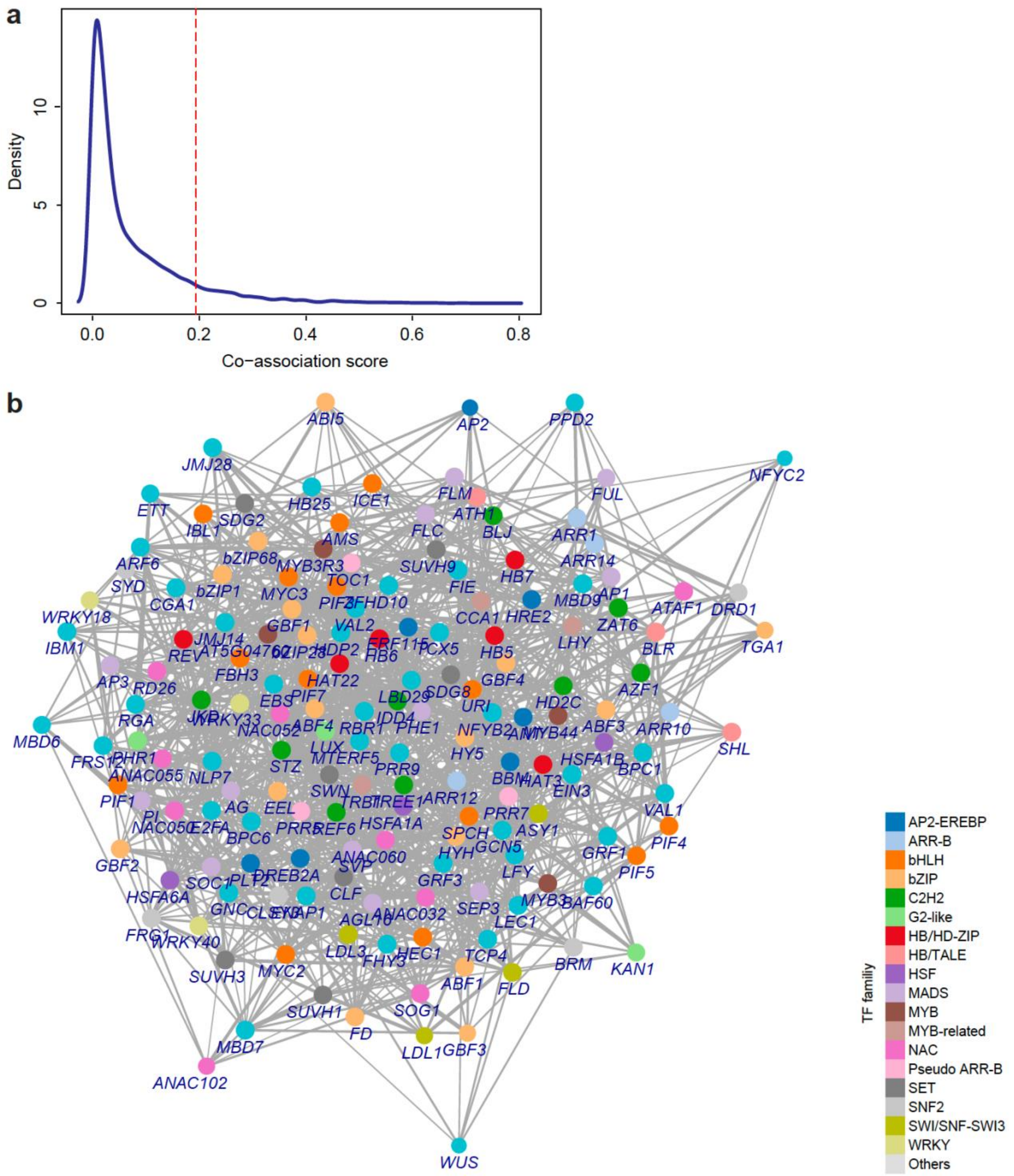

Supplementary Fig. 9. Co-associations of TFs, related to Fig. 3. (a) Determining an optimal threshold (highlighted in red) of significant TF co-associations based on an elbow statistic. **(b)** Network showing random co-associations between TFs. Co-association scores follow the same distribution as observed. However, no significant co-association modules were observed.

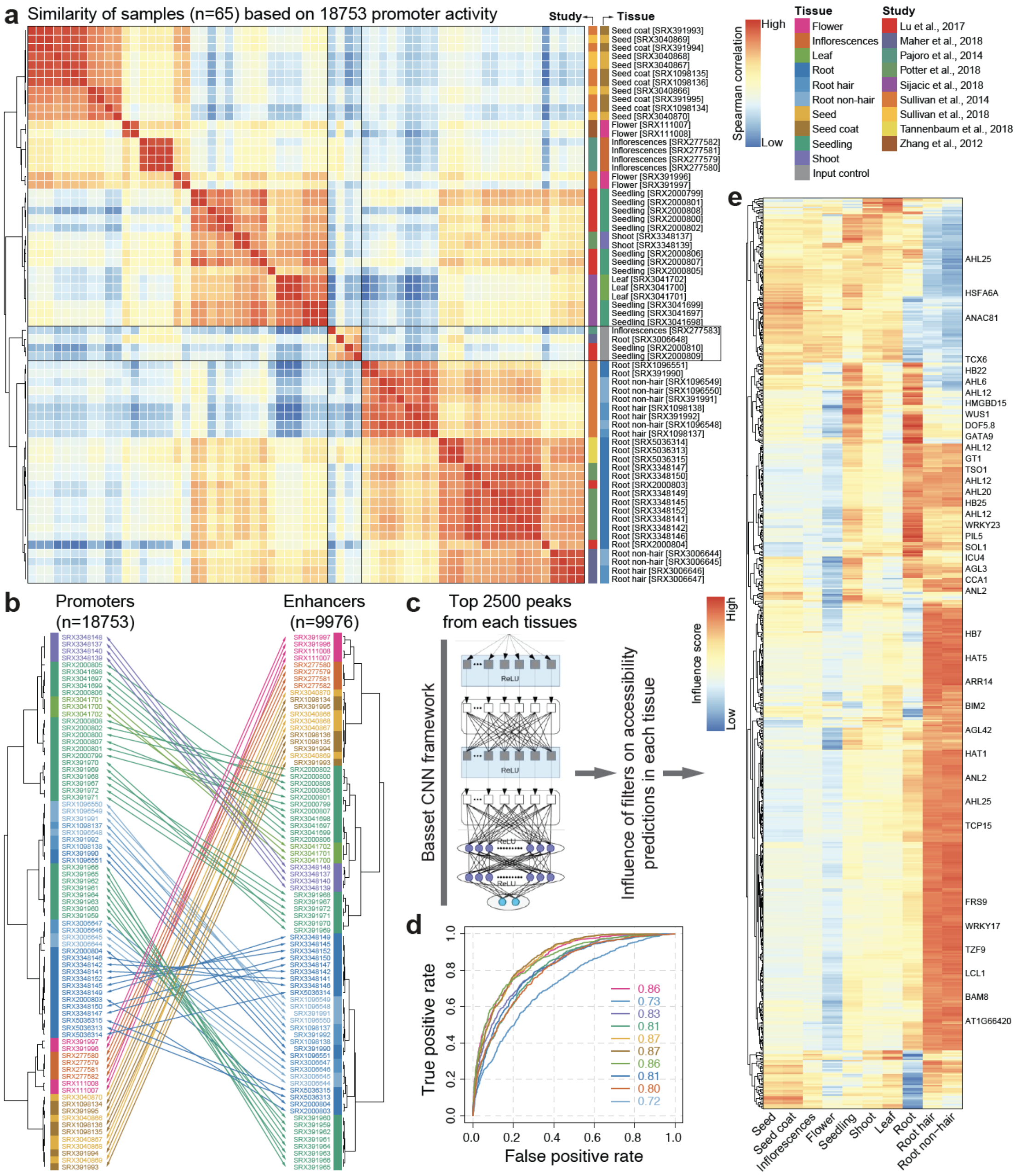

Supplementary Fig. 10. Dynamics of tissue-specific chromatin accessibility. (a) Sample similarity based on promoter activity. Open chromatin samples (with IDs labeled in square brackets) were collected from nine different studies. The input DNA samples (in grey; n=4) are used for control. **(b)** Comparing the clustering analysis based on enhancers (as in Fig. 4a) and promoters (as in a). **(c)** Predicting the sequence grammar underlying the chromatin dynamics of tissue-specific regulatory elements using the Basset[5] convolutional neural network (CNN) framework. **(d)** The ROC curves display the Basset false-positive rate versus true-positive rate for different tissues. **(e)** Heatmap showing the normalized influence of motif-annotated filters on the classification of enhancers in different tissues. Filters matched to known motifs

are labeled.

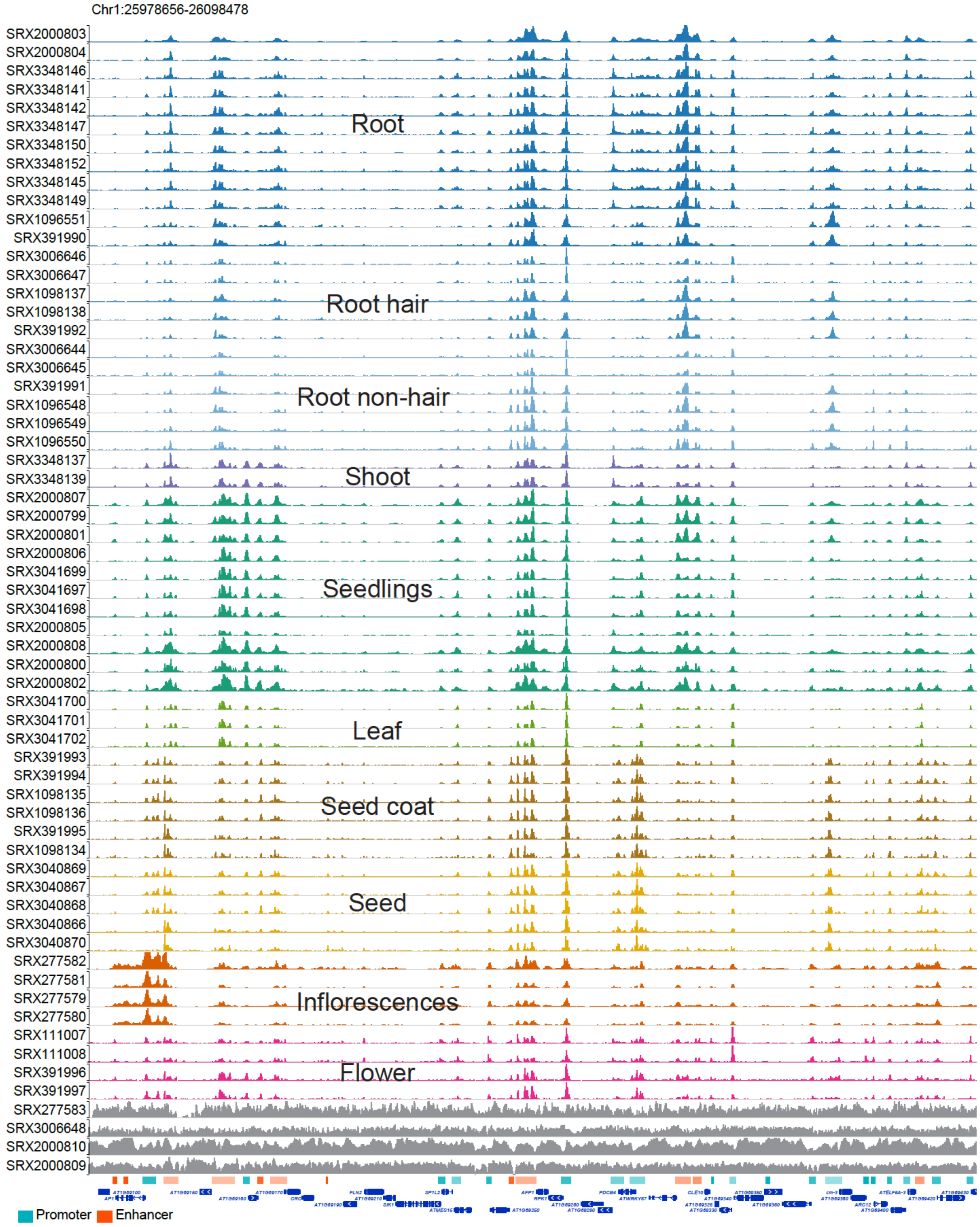

Supplementary Fig. 11. Tissue-specific regulatory elements (promoters and enhancers), related to Fig. 4. Genome browser view of open chromatin samples (colored as Fig. 4a). Annotated promoters and enhancers are provided at the bottom of the tracks.

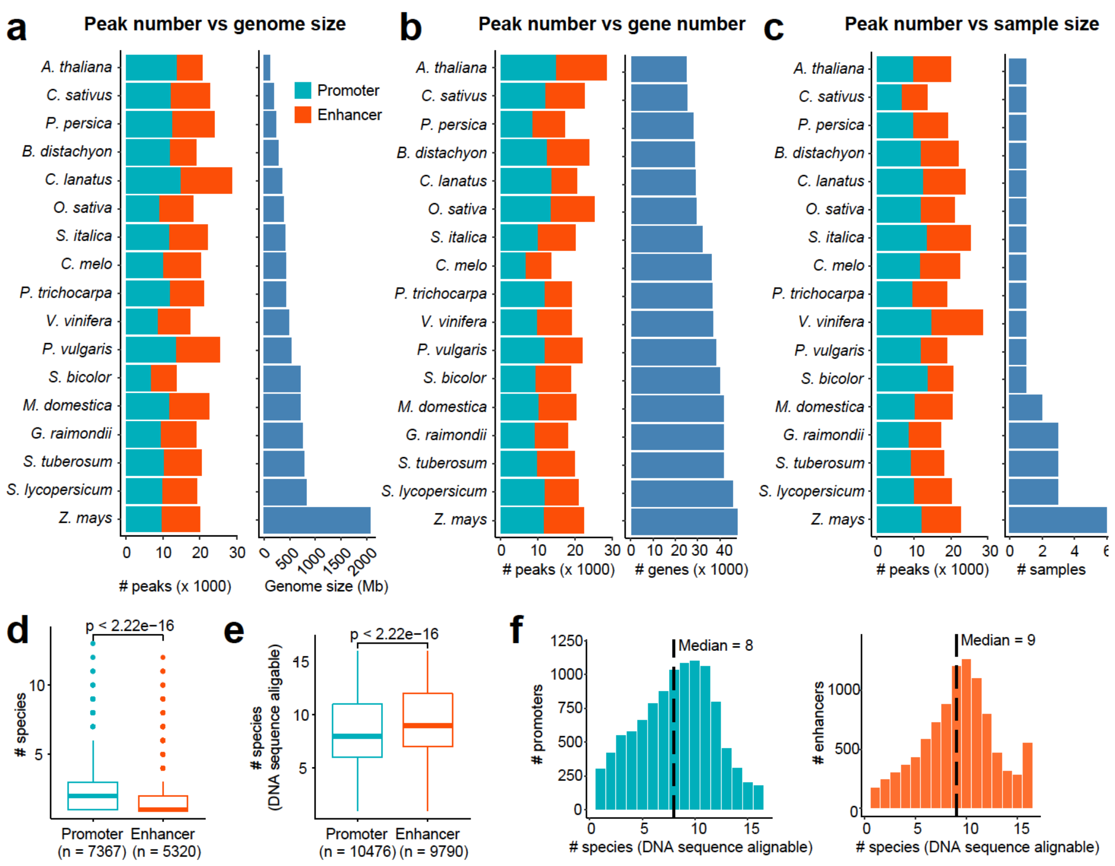

Supplementary Fig. 12. Quality control of promoter and enhancer evolution analysis. (a) The numbers of predicted promoters and enhancers per species are represented as stacked barplots in the left plot, ordered by the genome size for each species (right plot). The plots show that the genome size has little influence on the number of active regulatory regions identified per species. **(b)** As in (a), but numbers of promoters/enhancers in each species are ordered by the number of annotated genes in the corresponding genome. Gene numbers do not appear to influence variation in the number of promoters or enhancers identified in each species. **(c)** As in (a), but numbers of promoters/enhancers in each species are now ordered by the number of datasets used in the analysis. **(d)** Boxplot showing the number of species in which enhancer and promoter elements are conserved. Promoters are more conserved than enhancers. Statistical significance of difference was calculated by the two-sided Mann-Whitney U test. Boxplot shows the median (horizontal line), second to third quartiles (box), and Tukey-style whiskers (beyond the box). **(e)** The DNA sequences underlying promoters can be aligned to significantly lower numbers of species than the DNA sequences underlying enhancers, suggesting that higher conservation of promoter activity than enhancers is not due to differences in alignability. **(f)** Related to (e), barplots show the distribution of sequence alignability of promoters (left) and enhancers (right). The median numbers are shown.

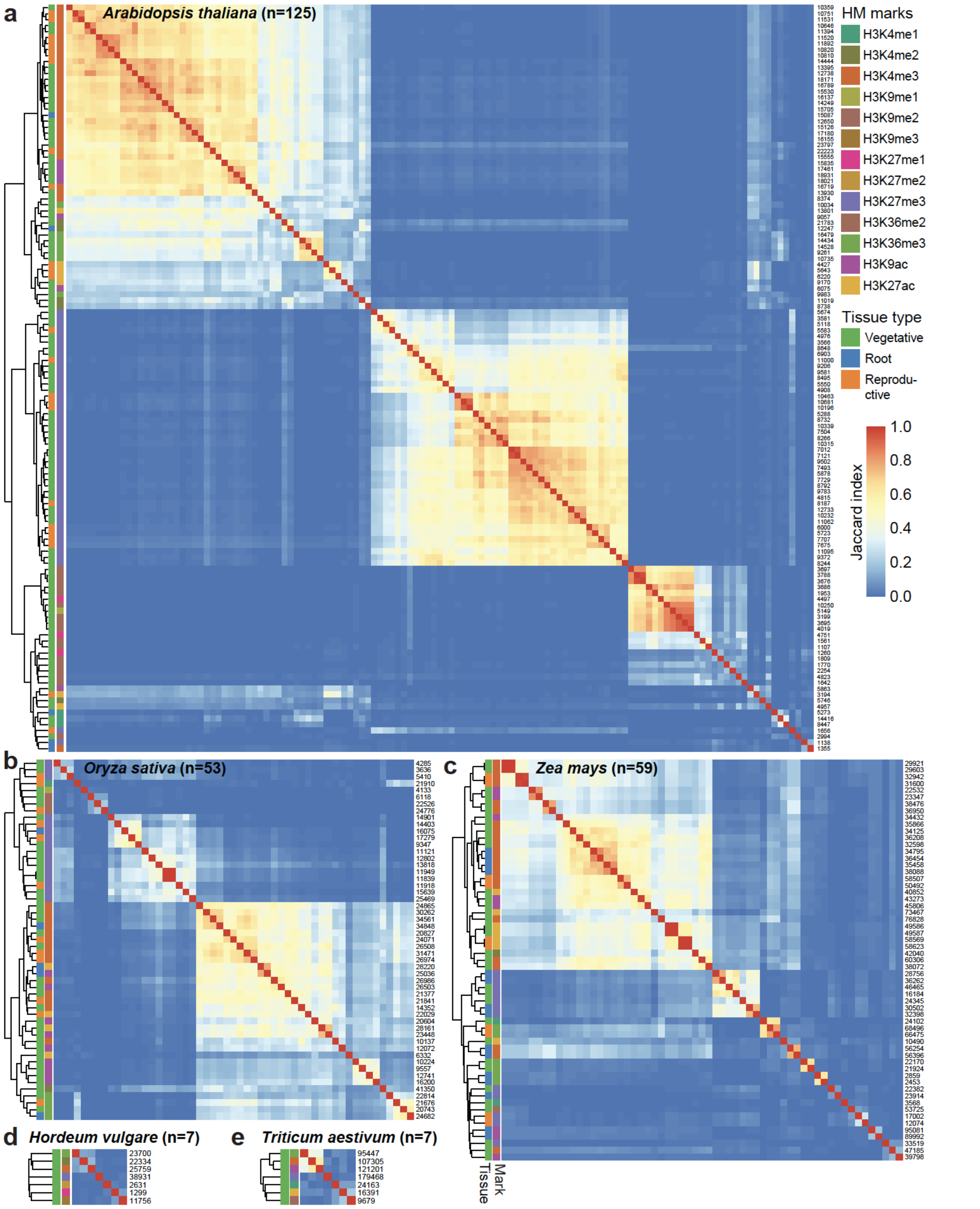

Supplementary Fig. 13. Manually curated histone modification ChIP-seq experiments used for ChromHMM. Heatmaps showing the similarity of histone modification ChIP-seq experiments in *Arabidopsis thaliana* (**a**), rice (*Oryza sativa*; **b**), maize (*Zea mays*; **c**), barley (*Hordeum vulgare*; **d**), and wheat (*Triticum aestivum*; **e**). The pairwise correlation of any two experiments were calculated based on the Jaccard index. Each experiment was assigned to one of the reference tissues (vegetative-, reproductive- and root-related tissues). Experiments are colored according to the type of tissues or marks (as indicated in the bar on the left of heatmaps). The number of peaks in each experiment is shown on the right of the heatmaps. The full list of

experiments can be found in Supplementary Data 9.

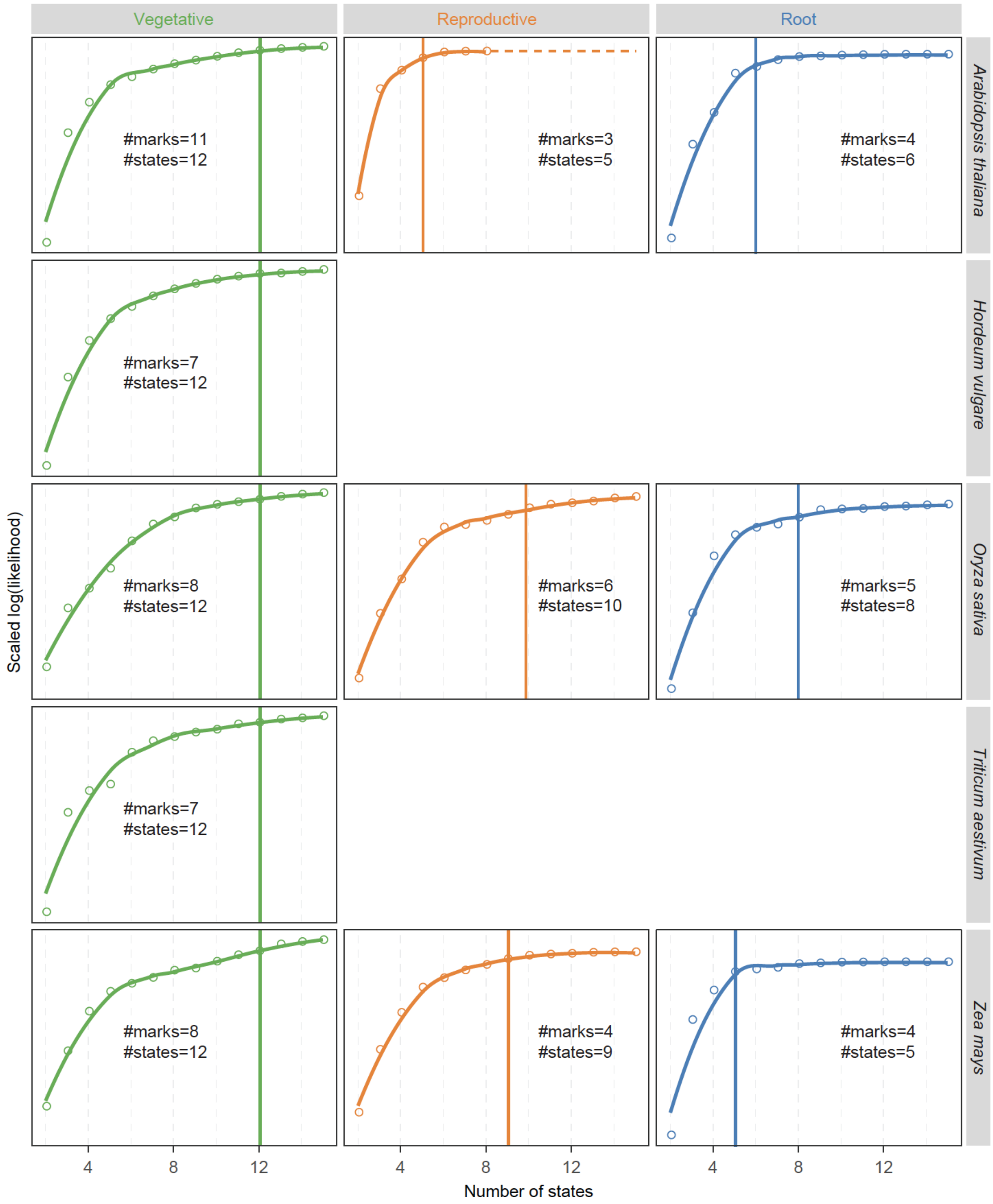

Supplementary Fig. 14. Chromatin states determined by ChromHMM. The ChromHMM[6] model was learned with up to 15 states for each reference tissue type (column) in each genome (row). The log(likelihood) of the model output by the program increased as the number of states increased, while the extent of increment declined after a specific number of states (dependent on tissue types and genomes) and the model was considered an “optimal” when reaching this number of states. The number of histone modification marks and the optimal number of chromatin states was indicated.

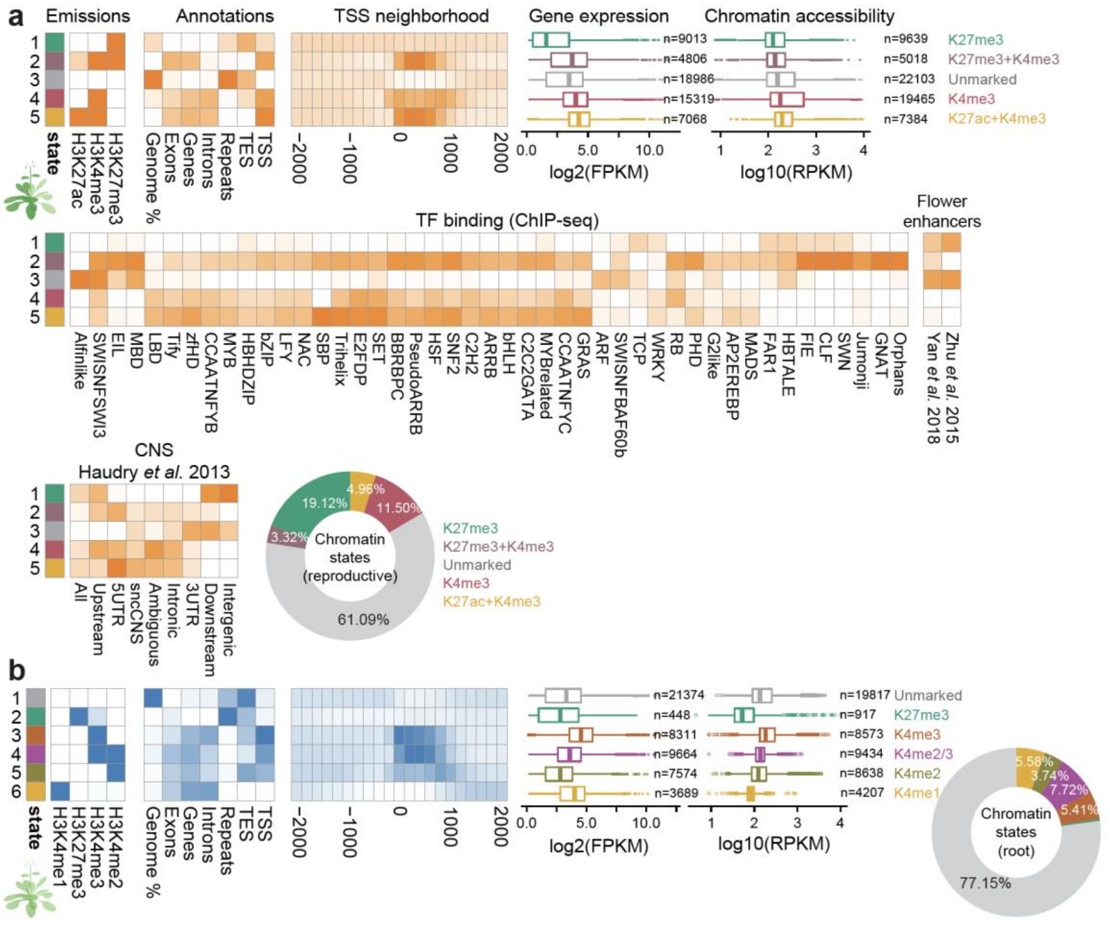

Supplementary Fig. 15. Chromatin states in Arabidopsis reproductive-related and root-related tissues. Prediction of chromatin states by ChromHMM6 in Arabidopsis reproductive-related **(a)** and root-related tissues **(b)**. In each figure, the left panel displays a heatmap of the emission parameters in which each row corresponds to a different state and each column corresponds to a histone modification mark; the second heatmap displays the overlap fold enrichment for different genomic annotations; the third heatmap shows the fold enrichment for each state for each 200-bp bin position within 2 kb around a set of transcription start sites (TSSs); Expression patterns for neighboring genes in each state are shown in the first boxplot; chromatin accessibility is shown in the second boxplot; the right donut chart shows the fraction of the entire genome covered by each of the states as shown on the left heatmaps. The “Unmarked” state is always shown in grey. TF binding and flower-related enhancers[1,7] are shown for each state in reproductive-related tissues. Gene expression and chromatin accessibility data in matched tissues from ref.[8]. Boxplots show the median (horizontal line), second to third quartiles (box), and Tukey-style whiskers (beyond the box). The number of genes or peaks in each category is indicated beside the boxplot.

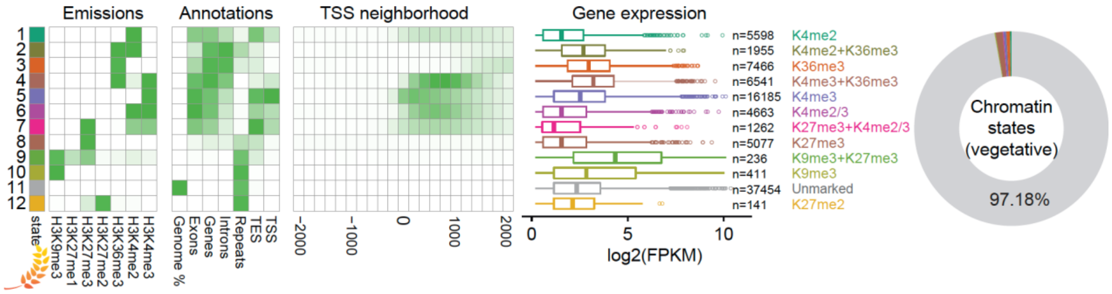

Supplementary Fig. 16. Chromatin states in barley (*Hordeum vulgare*). Prediction of chromatin states by ChromHMM in barley vegetative-related (a) tissue. The left panel displays a heatmap of the emission parameters in which each row corresponds to a different state and each column corresponds to a histone modification mark; the second heatmap displays the overlap fold enrichment for different genomic annotations; the third heatmap shows the fold enrichment for each state for each 200-bp bin position within 2 kb around a set of transcription start sites (TSSs); Expression patterns for neighboring genes in each state are shown in the first boxplot; he right donut chart shows the fraction of the entire genome covered by each of the states as shown on the left heatmaps.

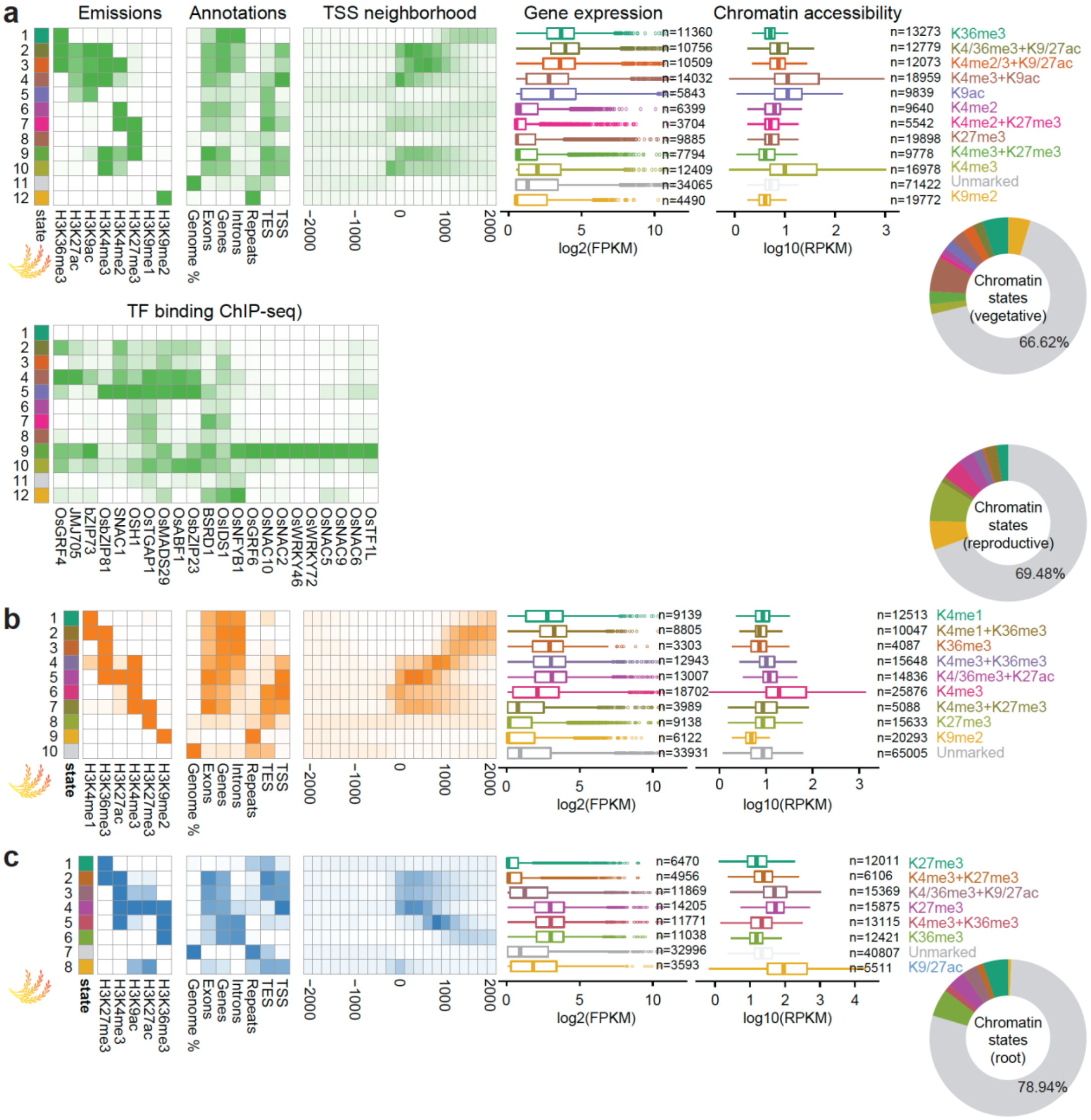

Supplementary Fig. 17. Chromatin states in rice (*Oryza sativa*). Prediction of chromatin states by ChromHMM in rice vegetative-related (a), reproductive-related (b) and root-related tissues (c). In each figure, the left panel displays a heatmap of the emission parameters in which each row corresponds to a different state and each column corresponds to a histone modification mark; the second heatmap displays the overlap fold enrichment for different genomic annotations; the third heatmap shows the fold enrichment for each state for each 200-bp bin position within 2 kb around a set of transcription start sites (TSSs); Expression patterns for neighboring genes in each state are shown in the first boxplot; chromatin accessibility is shown in the second boxplot; the right donut chart shows the fraction of the entire genome covered by each of the states as shown on the left heatmaps. "Unmarked" state is always shown in grey. TF binding is shown for each state in vegetative-related tissues.

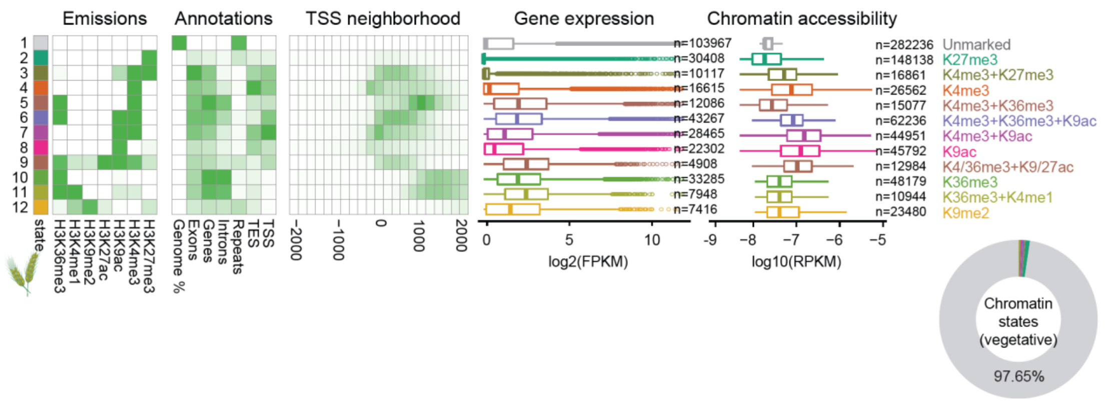

Supplementary Fig. 18. Chromatin states in wheat (*Triticum aestivum*). Prediction of chromatin states by ChromHMM in wheat vegetative-related (a) tissue. The left panel displays a heatmap of the emission parameters in which each row corresponds to a different state and each column corresponds to a histone modification mark; the second heatmap displays the overlap fold enrichment for different genomic annotations; the third heatmap shows the fold enrichment for each state for each 200-bp bin position within 2 kb around a set of transcription start sites (TSSs); Expression patterns for neighboring genes in each state are shown in the first boxplot; he right donut chart shows the fraction of the entire genome covered by each of the states as shown on the left heatmaps.

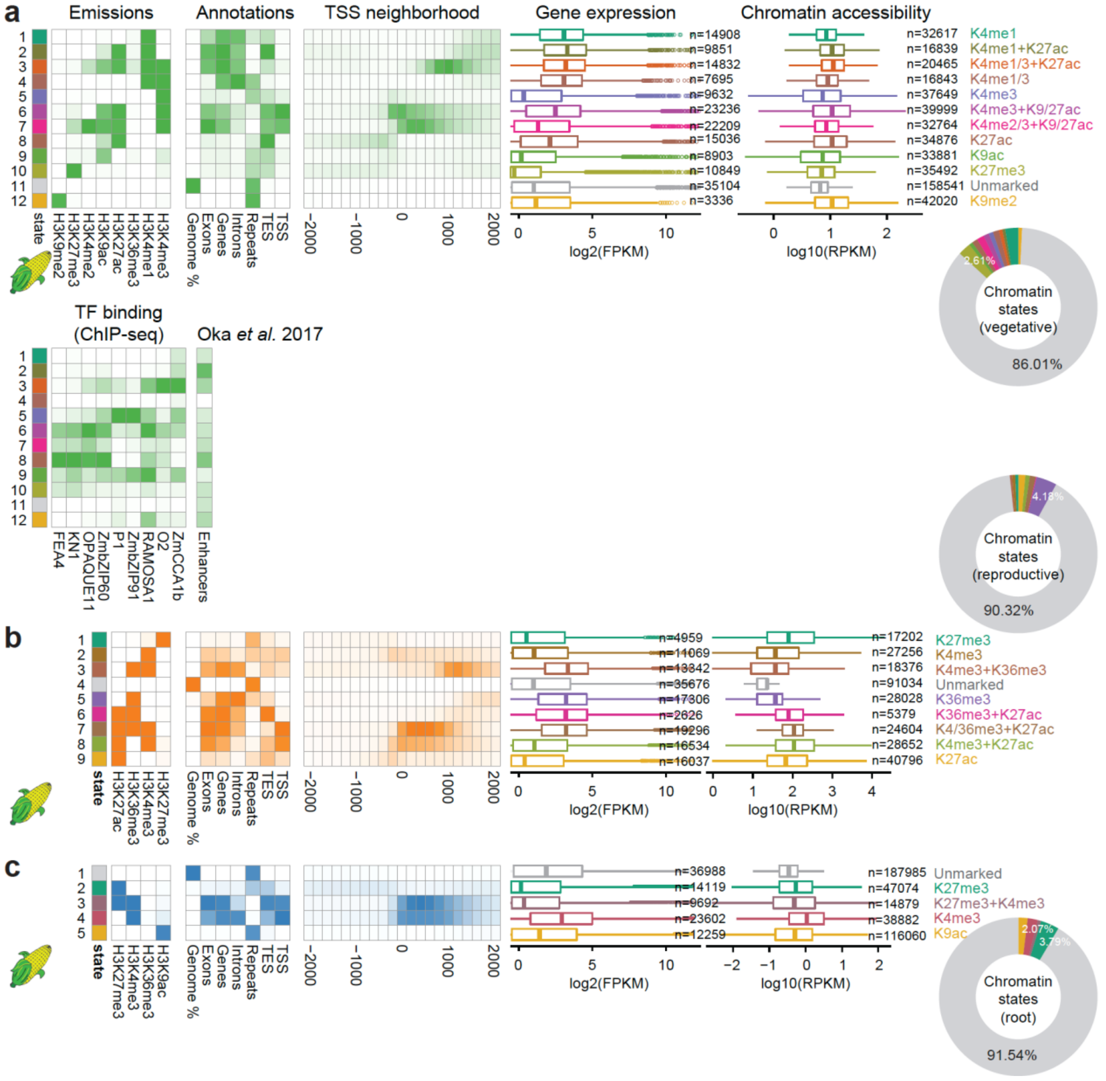

Supplementary Fig. 19. Chromatin states in maize (*Zea mays*). Prediction of chromatin states by ChromHMM in maize vegetative-related (a), reproductive-related (b) and root-related tissues (c). In each figure, the left panel displays a heatmap of the emission parameters in which each row corresponds to a different state and each column corresponds to a histone modification mark; the second heatmap displays the overlap fold enrichment for different genomic annotations; the third heatmap shows the fold enrichment for each state for each 200-bp bin position within 2 kb around a set of transcription start sites (TSSs); Expression patterns for neighboring genes in each state are shown in the first boxplot; chromatin accessibility is shown in the second boxplot; the right donut chart shows the fraction of the entire genome covered by each of the states as shown on the left heatmaps. “Unmarked” state is always shown in grey. TF binding and predicted enhancers from ref.[9] are shown for each state in vegetative-related tissues.

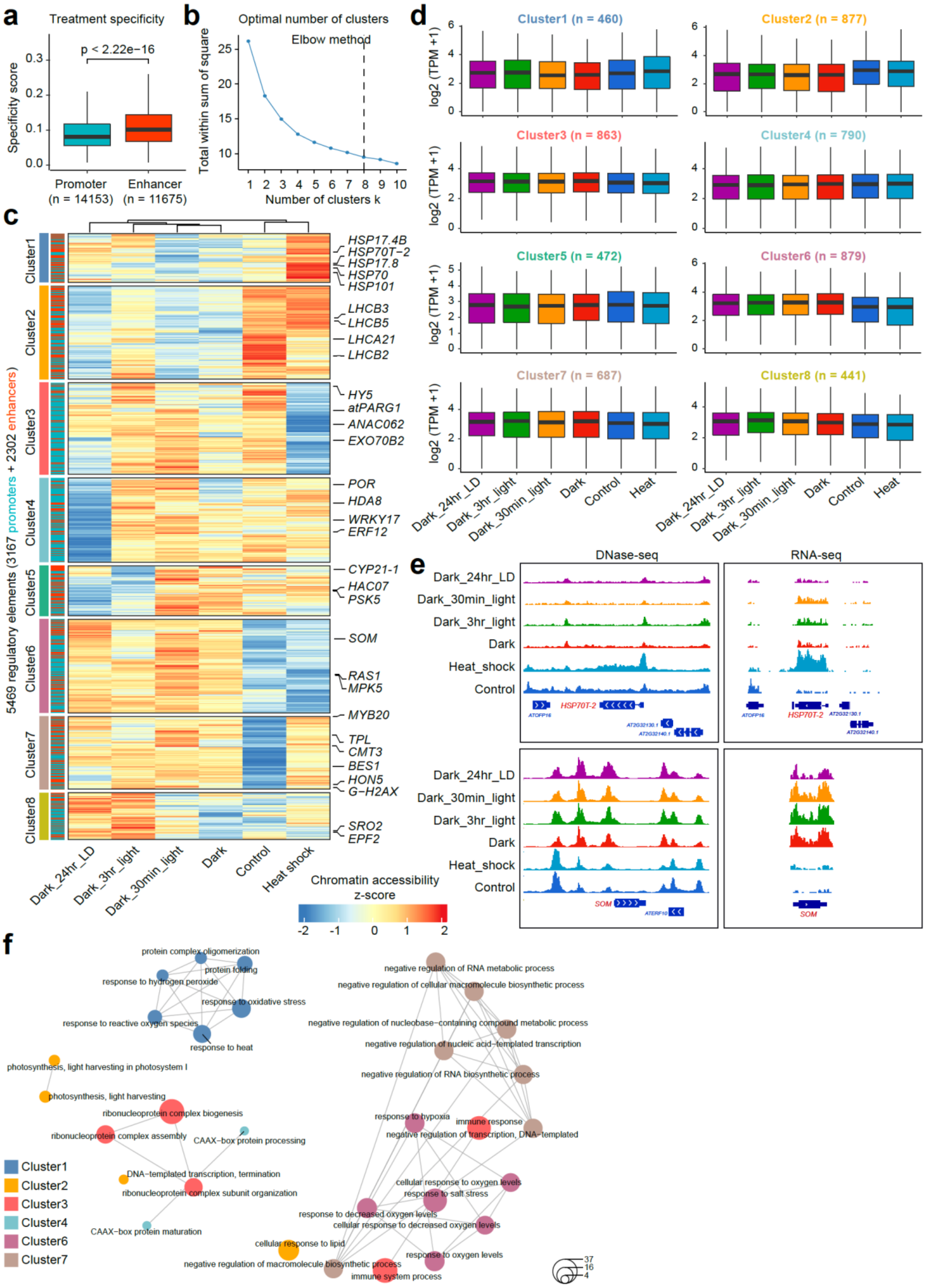

Supplementary Fig. 20. Dynamic activity of regulatory elements upon different stress treatments. DNase-seq data were taken from ref.8. **(a)** The boxplot showing the specificity score of promoter and enhancer accessibility. All peaks (n=25,828) were included. (b-c) Analysis of dynamically accessible peaks (n=5,469) upon stress treatments. The cutoff of peak treatment specificity was set as Tau index < 0.05 or > 0.2. **(b)** The optimal number (n=8) of clusters is determined by an elbow method. **(c)**

Heatmap showing the chromatin accessibility of 5469 highly specific regulatory elements (including promoters and enhancers). Regulatory elements are grouped into eight clusters based on their activity. Selected target genes are labeled on the right. **(d)** Boxplot showing the expression pattern of target genes in different clusters as in (b). The gene expression data was downloaded from the Arabidopsis RNA-seq database (http://ipf.sustech.edu.cn/pub/athrdb/)[10]. **(e)** Genome browser views of treatment-specific chromatin accessibility and RNA-seq read intensity at the two chosen gene loci: HSP70T-2 (ref.[11]) and SOMNUS (SOM)[12]. **(f)** Network plot showing a representative of enriched GO terms for target genes in different clusters. Only significantly enriched (adjusted p-value < 0.01) GO terms were shown. The circle size represents the number of genes belonging to a specific term. Boxplots show the median (horizontal line), second to third quartiles (box), and Tukey-style whiskers (beyond the box).

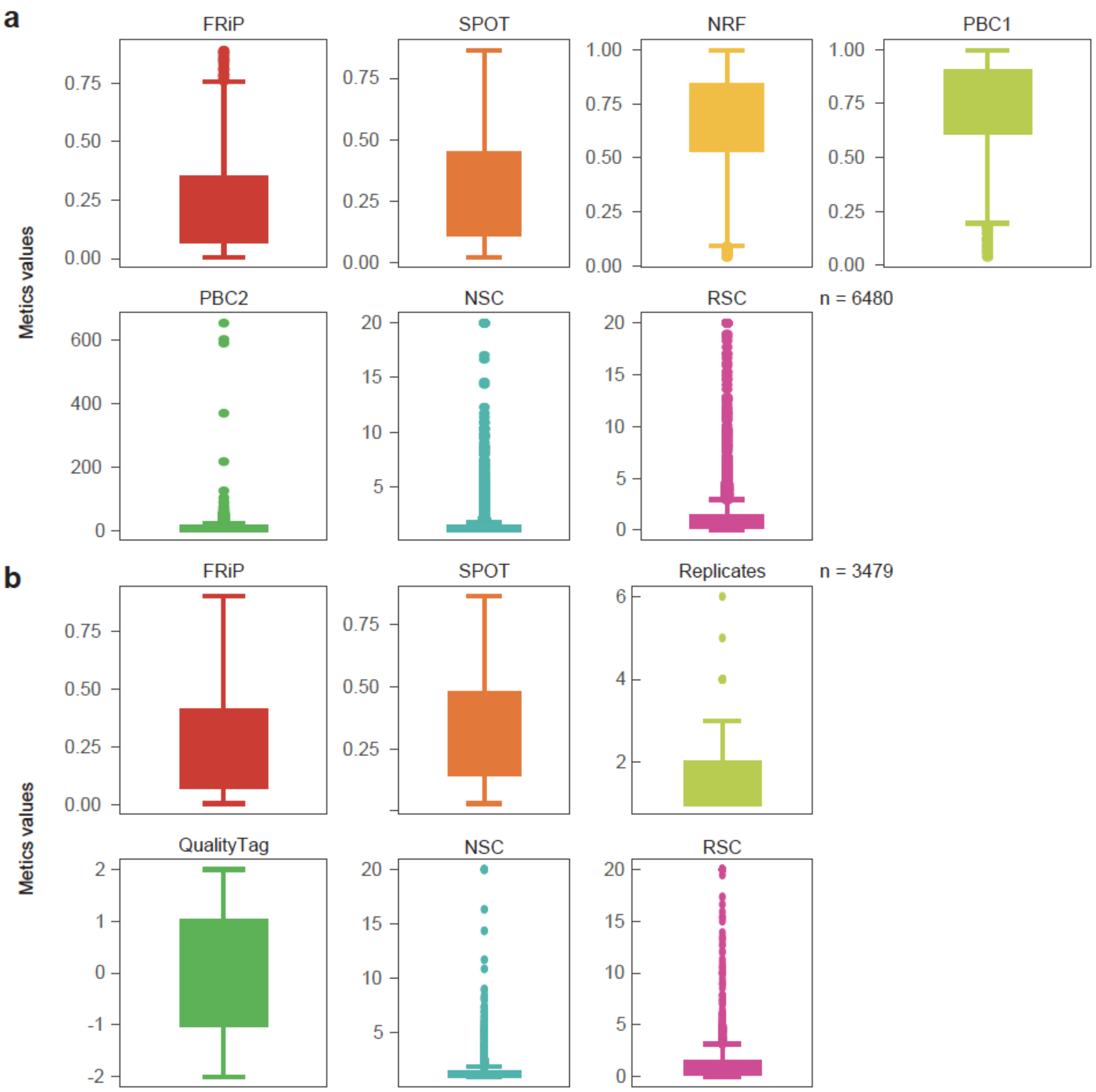

Supplementary Fig. 21. Quality metrics for plant regulome data. Various quality metrics for datasets/samples **(a)** and experiments **(b)** in Arabidopsis thaliana. Similar plots for other plant species can be drawn via the ChIP-Hub online tool. SPOT: signal portion of tags; FRiP: fraction of reads in peaks; NSC: normalized strand cross-correlation coefficient. RSC: relative Strand cross-correlation coefficient; NRF: non-redundant fraction; PBC1/2: PCR bottlenecking coefficients 1/2. Boxplots show the median (horizontal line), second to third quartiles (box), and Tukey-style whiskers (beyond the box).

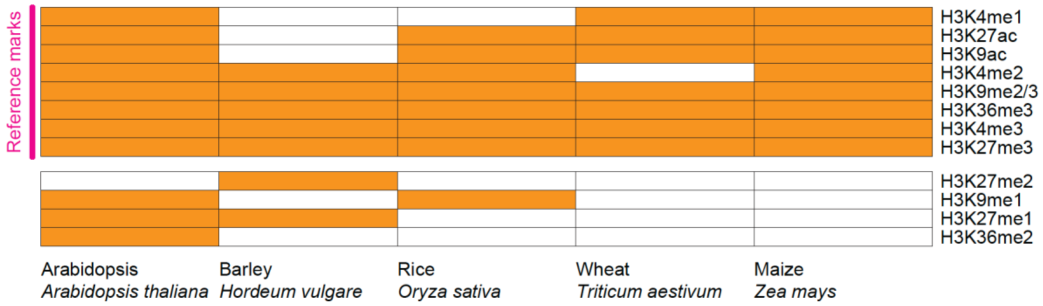

Supplementary Fig. 22. Types of histone modification marks used for chromatin state annotation in different plant species. Overview of histone modifications with available ChIP-seq data for chromatin state annotation in different plant species. A common set of marks (called “reference marks”) available in most (if not all) species are used for the comparison of chromatin states annotated in different species.

Supplementary references

1. Yan, W., Chen, D. & Kaufmann, K. Molecular mechanisms of floral organ specification by MADS domain proteins. *Curr Opin Plant Biol* **29**, 154–162 (2016).

2. Mateos, J. L. *et al*. Combinatorial activities of *SHORT VEGETATIVE PHASE and FLOWERING LOCUS C* define distinct modes of flowering regulation in Arabidopsis. *Genome Biol* **16**, 31 (2015).

3. Posé, D. *et al*. Temperature-dependent regulation of flowering by antagonistic FLM variants. *Nature* **503**, 414 (2013).

4. Lee, J. H. *et al*. Regulation of temperature-responsive flowering by MADS-box transcription factor repressors. *Science* **342**, 628–632 (2013).

5. Kelley, D. R., Snoek, J. & Rinn, J. L. Basset: learning the regulatory code of the accessible genome with deep convolutional neural networks. *Genome Res* **26**, 990–999 (2016).

6. Ernst, J. & Kellis, M. ChromHMM: automating chromatin-state discovery and characterization. *Nat Methods* **9**, 215 (2012).

7. Zhu, B., Zhang, W., Zhang, T., Liu, B. & Jiang, J. Genome-wide prediction and validation of intergenic enhancers in Arabidopsis using open chromatin signatures. *Plant Cell* **27**, 2415–2426 (2015).

8. Sullivan, A. M. *et al*. Mapping and dynamics of regulatory DNA and transcription factor networks in *A. thaliana*. *Cell Reports* **8**, 2015–2030 (2014).

9. Oka, R. *et al*. Genome-wide mapping of transcriptional enhancer candidates using DNA and chromatin features in maize. *Genome Biol* **18**, 137 (2017).

10. Zhang, H. *et al*. A comprehensive online database for exploring 20,000 public Arabidopsis RNA-seq libraries. *Mol Plants* **13**, 1231–1233 (2020).

11. Lin, B.-L. *et al*. Genomic analysis of the Hsp70 superfamily in *Arabidopsis thaliana*. *Cell Stress Chaperones* **6**, 201-208 (2001).

12. Kim, D. H. *et al*. SOMNUS, a CCCH-type zinc finger protein in Arabidopsis, negatively regulates light-dependent seed germination downstream of PIL5. *Plant Cell* **20**, 1260–1277 (2008).
