# Supplementary material for: ChIP-Hub provides an integrative platform for exploring plant regulome
Source: Nat Commun. 2022 Jun 14;13:3413. doi: 10.1038/s41467-022-30770-1 (PMC9197862; doi:10.1038/s41467-022-30770-1)
Supplement: Supplementary file 3 — Description of Additional Supplementary Files [file 41467_2022_30770_MOESM3_ESM.pdf]

Description of Additional Supplementary Files

Supplementary Data 1

Description: Number of identified peaks in all experiments.

Supplementary Data 2

Description: ChIP-seq experiments in Arabidopsis thaliana used in the analysis.

Supplementary Data 3

Description: TF-miRNA-TF FFLs in Arabidopsis thaliana.

Supplementary Data 4

Description: Open chromatin datasets used for enhancer prediction.

Supplementary Data 5

Description: Quantitative analysis of promoters and enhancers.

Supplementary Data 6

Description: GO analysis of tissue-specific regulatory elements. P-values are calculated by the hypergeometric distribution test and adjusted by the Benjamini-Hochberg method.

Supplementary Data 7

Description: Open chromatin data used for comparitive genome analysis.

Supplementary Data 8

Description: Promoters and Enhancers identified in this study.

Supplementary Data 9

Description: Histone modification ChIP-seq experiments used for ChromHMM analysis.

Supplementary Data 10

Description: Examples of metadata files. Different cases of experiment metadata.

Supplementary Data 11

Description: Plant species and genome information used in this study.

Supplementary Data 12

Description: Quality categories for different metrics.
