# Supplementary material for: ChIP-Hub provides an integrative platform for exploring plant regulome
Source: Nat Commun. 2022 Jun 14;13:3413. doi: 10.1038/s41467-022-30770-1 (PMC9197862; doi:10.1038/s41467-022-30770-1)
Supplement: Supplementary file 16 — Reporting Summary [file 41467_2022_30770_MOESM16_ESM.pdf]

nature portfolio

Corresponding author(s): Dijun Chen

Last updated by author(s): May 16, 2022

Reporting Summary

Nature Portfolio wishes to improve the reproducibility of the work that we publish. This form provides structure for consistency and transparency in reporting. For further information on Nature Portfolio policies, see our Editorial Policies and the Editorial Policy Checklist.

Statistics

For all statistical analyses, confirm that the following items are present in the figure legend, table legend, main text, or Methods section.

n/a	Confirmed	
☐	☒	The exact sample size (*n*) for each experimental group/condition, given as a discrete number and unit of measurement
☒	☐	A statement on whether measurements were taken from distinct samples or whether the same sample was measured repeatedly
☐	☒	The statistical test(s) used AND whether they are one- or two-sided<br>*Only common tests should be described solely by name; describe more complex techniques in the Methods section.*
☒	☐	A description of all covariates tested
☒	☐	A description of any assumptions or corrections, such as tests of normality and adjustment for multiple comparisons
☐	☒	A full description of the statistical parameters including central tendency (e.g. means) or other basic estimates (e.g. regression coefficient) AND variation (e.g. standard deviation) or associated estimates of uncertainty (e.g. confidence intervals)
☐	☒	For null hypothesis testing, the test statistic (e.g. *F*, *t*, *r*) with confidence intervals, effect sizes, degrees of freedom and *P* value noted<br>*Give P values as exact values whenever suitable.*
☒	☐	For Bayesian analysis, information on the choice of priors and Markov chain Monte Carlo settings
☐	☒	For hierarchical and complex designs, identification of the appropriate level for tests and full reporting of outcomes
☒	☐	Estimates of effect sizes (e.g. Cohen's *d*, Pearson's *r*), indicating how they were calculated

Our web collection on statistics for biologists contains articles on many of the points above.

Software and code

Policy information about availability of computer code

Data collection	SRA Toolkit (version 2.5.1)

Data analysis	Trim Galore program (version 0.4.1)<br>FastQC (version 0.11.8)<br>Bowtie2 (version 2.2.6)<br>Picard tools (version 2.60; http://broadinstitute.github.io/picard/)<br>SAMtools (version 0.1.19)<br>MACS2 (version 2.1.0)<br>phantompeakqualtools package (https://code.google.com/p/phantompeakqualtools/)<br>BEDTools (version 2.25.0)<br>DeepTools (version 3.5.0)<br>WashU Epigenome Browser (version 46.2)<br>BLAST (version 2.9.0)<br>FIMO (version 5.4.1)<br>ChromHMM (version 1.19)<br>LastZ (version 1.04.03)<br>Basset (https://github.com/davek44/Basset)<br>Shiny (version 1.5.12.933)<br>ggplot2 (vsrsion 3.3.3)<br>R (version 3.4.1)<br>Hotspot (HotSpot5)

For manuscripts utilizing custom algorithms or software that are central to the research but not yet described in published literature, software must be made available to editors and reviewers. We strongly encourage code deposition in a community repository (e.g. GitHub). See the Nature Portfolio guidelines for submitting code & software for further information.

Data

Policy information about availability of data

All manuscripts must include a data availability statement. This statement should provide the following information, where applicable:

- Accession codes, unique identifiers, or web links for publicly available datasets
- A description of any restrictions on data availability
- For clinical datasets or third party data, please ensure that the statement adheres to our policy

Raw data and related annotation information were obtained from NCBI SRA (https://www.ncbi.nlm.nih.gov/sra), GEO (https://www.ncbi.nlm.nih.gov/geo), European Nucleotide Archive (ENA, https://www.ebi.ac.uk/ena), PlantTFDB (http://planttfdb.gao-lab.org/quick_search_result.php), JASPAR (https://jaspar.genereg.net/) and CIS-BP (http://cisbp.ccbr.utoronto.ca/). ChIP-Hub is available at https://biobigdata.nju.edu.cn/ChIPHub/. Users can view the processed data through the Epigenome Browser https://biobigdata.nju.edu.cn/browser/. All the analysis results (including peak files in the BED format, signal files in the bigwig format, comparative genomics data, predicted promoters/enhancers and gene regulatory networks) can be downloaded through the link https://biobigdata.nju.edu.cn/ChIPHub_download/. Metadata and peak files (in the BED format) for all curated experiments in the current version of ChIP-Hub are also deposited at Zenodo (doi: https://doi.org/10.5281/zenodo.5912234). Source Data are provided with this paper.

Field-specific reporting

Please select the one below that is the best fit for your research. If you are not sure, read the appropriate sections before making your selection.

☒ Life sciences ☐ Behavioural & social sciences ☐ Ecological, evolutionary & environmental sciences

For a reference copy of the document with all sections, see nature.com/documents/nr-reporting-summary-flat.pdf

Life sciences study design

All studies must disclose on these points even when the disclosure is negative.

Sample size	This study collected >10000 publicly available datasets for >40 plant species from ~540 original publications.
Data exclusions	All downloaded data were included in the analysis.
Replication	All the data analysis are provided the related codes to ensure its reproducibility. All attempts to repeat bioinformatic analyses were successful.
Randomization	This research does not involve randomization or grouping of samples.
Blinding	None of analysis could be influenced at any stage nor did they have known a priori outcomes. No additional blinding measures were required.

Reporting for specific materials, systems and methods

We require information from authors about some types of materials, experimental systems and methods used in many studies. Here, indicate whether each material, system or method listed is relevant to your study. If you are not sure if a list item applies to your research, read the appropriate section before selecting a response.

Materials & experimental systems

n/a	Involved in the study
☒	☐ Antibodies
☒	☐ Eukaryotic cell lines
☒	☐ Palaeontology and archaeology
☒	☐ Animals and other organisms
☒	☐ Human research participants
☒	☐ Clinical data
☒	☐ Dual use research of concern

Methods

n/a	Involved in the study
☒	☐ ChIP-seq
☒	☐ Flow cytometry
☒	☐ MRI-based neuroimaging
